# Potassium channels in animal models of post-traumatic stress disorder: mechanistic and therapeutic implications

**DOI:** 10.3389/fncel.2024.1441514

**Published:** 2024-07-30

**Authors:** Ravi Philip Rajkumar

**Affiliations:** Department of Psychiatry, Jawaharlal Institute of Postgraduate Medical Education and Research, Pondicherry, India

**Keywords:** post-traumatic stress disorder, potassium channels, HCN1, KCNQ, GIRK, amygdala, medial prefrontal cortex

## The significance of potassium channels in neuropsychiatric disorders

Potassium (K^+^) channels are a diverse group of ion channels which regulate the excitability and stability of biological membranes through their effects on inward K^+^ flux, leading to reduced excitability and hyperpolarization. Contemporary classifications recognize anywhere from three to five subtypes of K^+^ channels, based on their subunit composition, number of transmembrane domains, and functional properties. The four most widely recognized subtypes are: (a) *voltage-gated* K channels (K_V_), (b) *calcium (Ca*++*)-activated* K channels (K_Ca)_, (c) *inward-rectifying* K channels (K_IR_), and (d) *two-pore domain* K channels (K_2P_). Apart from these, there are *ligand-gated* K channels that are activated by specific molecules, such as cyclic nucleotides (Kuang et al., 2015).

K^+^ channels are highly expressed in several brain regions, including the frontal cortex, basal ganglia, hippocampus and amygdala, where they influence neuronal firing, transmitter release, and neural plasticity. Mendelian disorders involving K^+^ channel-related mutations in the brain have been associated with developmental delays, epilepsy, and symptoms suggestive of anxiety, hyperactivity, and autism spectrum disorder (Alam et al., 2023). This has led researchers to investigate the possible contribution of K^+^ channel functioning to non-Mendelian psychiatric syndromes. Such research has found possible evidence of altered K^+^ channel activity in schizophrenia, depression, and autism spectrum disorders. This raises the possibility of novel therapeutic approaches aimed at modulating the functioning of these channels (Vukadinovic and Rosenzweig, 2012; Cheng et al., 2021; Meshkat et al., 2024). More recent research has highlighted the importance of K^+^ channels in anxiety- and fear-related processes. In animal models, K_V_ channels have been found to play a key role in fear conditioning and anxiety-like behaviors (Stubbendorff et al., 2023; Page and Coutellier, 2024). In humans, polymorphisms in genes encoding K_V_ and K_IR_ channel subunits have been associated with vulnerability to anxiety disorders in youth (Thapaliya et al., 2023). This paper examines recent translational evidence implicating changes in K^+^ channel functioning in the pathogenesis of post-traumatic stress disorder.

## Understanding the neurobiology of post-traumatic stress disorder

Post-traumatic stress disorder (PTSD) is a chronic psychiatric syndrome caused by exposure either to a single, overwhelming traumatic stressor or chronic traumatic stress. PTSD affects about 6–8% of the global population, and is characterized by intrusive “re-experiencing” of the traumatic event, increased arousal, avoidance of trauma-related cues, and associated changes in thought and mood, which can persist for months or years after a traumatic exposure. Currently approved treatments for PTSD include serotonergic antidepressants and specific types of psychotherapy, but their efficacy is often limited (Lee et al., 2016).

The neurobiology of PTSD is complex and involves dysregulation of neurotransmitter, neuroendocrine and immune-inflammatory pathways (Ressler et al., 2022). The core features of PTSD probably reflect dysregulation of fear-related processes, involving neural circuits connecting the ventromedial prefrontal and anterior cingulate cortices to the insula and limbic structures such as the hippocampus and the central and basolateral regions of the amygdala (France and Jovanovic, 2023). Distinct symptom domains of PTSD may reflect alterations in discrete neural circuits. For example, hypervigilance may reflect increased amygdala activity, while alterations in sleep may result from additional alterations in the functioning of the insula, hippocampus, and dorsal anterior cingulate cortex (Ressler et al., 2022).

This neuroanatomical diversity reflects a diversity in pathogenic cellular mechanisms. In a recent genome-wide association study (GWAS) of over 150,000 patients with PTSD, 43 genes coding for neurotransmitter receptors, ion channels, neural development, synaptic structure and function, and the regulation of endocrine and immune responses were all associated with vulnerability to this disorder. A common thread that unites this diverse set of genes is that they all are involved in fear-, threat- and stress-related psychophysiological responses (Nievergelt et al., 2024). A smaller GWAS of trauma-exposed adults found a suggestive association between a polymorphism of the *DPP6* gene and certain specific symptoms of PTSD, such as experiences of unreality and detachment from one's surroundings (Wolf et al., 2014). *DPP6* codes for a protein that is associated with a particular subtype of voltage-gated (K_V_) K^+^ channel, which is involved in the regulation of dendritic excitability and synaptic integration of information (Sun et al., 2011). This raises the possibility that K^+^ channel subtypes play a role in the development of specific PTSD symptoms, and may even represent potential therapeutic targets.

## Potassium channels in animal models of fear-related phenomena

Research involving rodent models of fear memory, conditioning and extinction has found that at least three types of K^+^ channels are involved in these processes. Kir3 channels, also known as GIRK channels, are tetrameric, G-protein-gated inwardly rectifying (K_IR_) K^+^ channels, which exist in four varieties labeled GIRK1 through GIRK4. GIRK1 and GIRK2 are more highly expressed in the brain. Activation of these channels with an experimental agent has been found to facilitate the extinction of conditioned fear responses in mice, probably through increased GIRK inhibitory tone in the basolateral amygdala (BLA) (Xu et al., 2020). GIRK channels can be activated by the neurotransmitter gamma-aminobutyric acid (GABA) through GABA_B_ receptors, and it has been found that GIRK-mediated currents in the prelimbic area of the medial prefrontal cortex are stronger in male than in female mice. These gender differences in GIRK-mediated fear extinction may account for the increased susceptibility of women to PTSD following exposure to trauma (Fernandez de Velasco et al., 2015).

KCNQ, a type of K_V_ K^+^ channel, appears to inhibit the consolidation of fear-related memories in the BLA. The use of a KCNQ agonist was associated with impaired fear consolidation in mice. Inhibition of the KCNQ-mediated current through direct antagonism, or through the activation of muscarinic M_1_, adrenergic β_2_, or dopaminergic D_5_ receptors, had the opposite effect, leading to enhanced fear consolidation. This suggests that monoamine transmitters may influence fear memory consolidation through their effects on this channel (Young and Thomas, 2014). In primates, activation of the α_1_ adrenergic receptor in the prefrontal cortex has complex effects: postsynaptic α_1_ receptors on dendritic spines lead to KCNQ opening and reduced cortical activity, while presynaptic α_1_ receptors increase cortical activity. It has been hypothesized that the former mechanism is operative at high levels of stress, leading to reduced cortical regulation of subcortical fear and stress responses (Datta et al., 2019). This may explain why the α_1_-receptor antagonist prazosin is effective in some patients with PTSD. More importantly, these results highlight the importance of considering the localization of K^+^ channels when evaluating their effects on fear-related disorders. In this case, KCNQ activation in the amygdala appears to protect against PTSD, but may have the opposite effect in the prefrontal cortex.

SK channels, which are K_Ca_-type K^+^ channels, may also have significant effects on fear conditioning in mice. More specifically, they may inhibit the activity of infralimbic cortical neurons involved in fear extinction. In rats undergoing fear conditioning, blocking SK channels had no immediate effect on fear responses, but increased the extinction of fear responses on the next day; activation of SK channels led to hyperpolarization of infralimbic neurons and reduced fear extinction (Criado-Marrero et al., 2014). A somewhat different picture was obtained in the mouse amygdala, where fear conditioning was associated with reduced SK2 channel expression in the BLA, while fear extinction was associated with increased numbers of synaptic SK2 channels, an effect which appeared to be mediated by the synaptic regulator protein membrane palmitoylated protein 2 (MPP2) (Peng et al., 2023). K_Ca_ channel activation may also inhibit the increased excitability of the lateral amygdala caused by chronic stress in rats (Rosenkranz et al., 2010). These results are remarkably similar to those observed with KCNQ. Overall, potassium channel activation in the BLA may inhibit the consolidation of fear memories, while activation of the same channels in adjacent regions of the medial prefrontal cortex may inhibit their extinction (Criado-Marrero et al., 2014).

## Potassium channels in animal models of PTSD

PTSD-like behavior can be induced in animals through exposure to experimental trauma, which involves prolonged immobilization and forced swimming (single prolonged stress, SPS) either alone or in combination with electric foot shock (single prolonged stress and shock, SPS&S) (Zhang et al., 2019). These animal models of PTSD have also been studied in relation to potential changes in K^+^ channel expression and functioning. Hyperpolarization-activated cyclic nucleotide-gated channel 1 (HCN1), a six-transmembrane domain K^+^ channel highly expressed in brain regions such as the frontal cortex and hippocampus (Zhao et al., 2023), was examined in rats exposed to SPS&S. It was found that inhibition of HCN1 alleviated PTSD-like behaviors, while administration of a HCN1 activator increased them. These behavioral changes appeared to be related to the brain-derived neurotrophic factor (BDNF)-mTOR signaling pathway, which is involved in synaptic plasticity, and activation of HCN1 appears to antagonize this pathway (Ni et al., 2020). In a separate study of mice, prenatal exposure to alcohol increased the likelihood of PTSD-like behavior in offspring exposed to electric foot shock. This susceptibility was associated with increased expression of HCN1 in the prefrontal cortex, but not the hippocampus, and administration of a HCN1 antagonist increased fear extinction and reduced PTSD- and depressive-like behavior (Yao et al., 2023). In an independent study of the SPS&S model of PTSD in rats, exposure to SPS&S was associated with increased expression of HCN1 and reduced expression of BDNF. Administration of ketamine, an antagonist of the N-methyl d-aspartate (NMDA) glutamate receptor, ameliorated PTSD-like behavior and led to reduced HCN1 expression and increased BDNF levels. Overall, a negative correlation was observed between prefrontal BDNF and HCN1 expression (Hou et al., 2018). Similar results were found in an SPS model of PTSD in mice, where the administration of ketamine reduced PTSD-like behavior and normalized stress-induced elevations in HCN1 in the prefrontal cortex, but not in the hippocampus (Zhang X. et al., 2021). The consistency and replicability of these findings suggest that prefrontal HCN1 K^+^ channel expression increases after traumatic stress, and may contribute to PTSD symptoms through an apparently antagonistic effects on the neurotrophic and neuroplasticity-enhancing effects of BDNF.

Two other K+ channel subtypes have been tentatively implicated in mouse models of PTSD. In the first, PTSD-like behavior and hippocampal expression of the K_IR_ channel Kir4.1 were reduced by the administration of a ginsenoside, and increased by intra-cerebroventricular injection of the pro-inflammatory cytokine TNFα in mice exposed to SPS (Zhang Z. et al., 2021). In the second, apparent improvements in PTSD-like behavior in an SPS&S model were observed with a polyherbal extract, and these beneficial changes were associated with increased phosphorylation of the K_V_ channel Kv4.2 in the hippocampus (Park et al., 2023). As these results have not yet been replicated, their significance is uncertain.

## Other possible links between potassium channels and PTSD

There are other indirect sources of evidence linking altered K+ channel functioning to PTSD, derived from research not directly involving animal models of this disorder. Increasing the expression of the outward rectifying K_V_ channel Kv1.1 through a viral vector is associated with reduced BLA firing and reduced hippocampal neurogenesis in rats, though the relevance of these changes to PTSD is uncertain (Kirby et al., 2012). Animals exposed to chronic stress exhibit increased K^+^ channel opening in the prefrontal cortex, which appears to be mediated by the activation of α_1_-adrenergic receptors and D_1_ dopamine receptors (Datta and Arnsten, 2019). Conversely, the “anti-stress” peptide transmitter neuropeptide Y (NPY) has been associated with activation of GIRK channels, leading to reduced activity of the BLA which may protect against the development of PTSD (Tasan et al., 2016).

**Figure 1:**
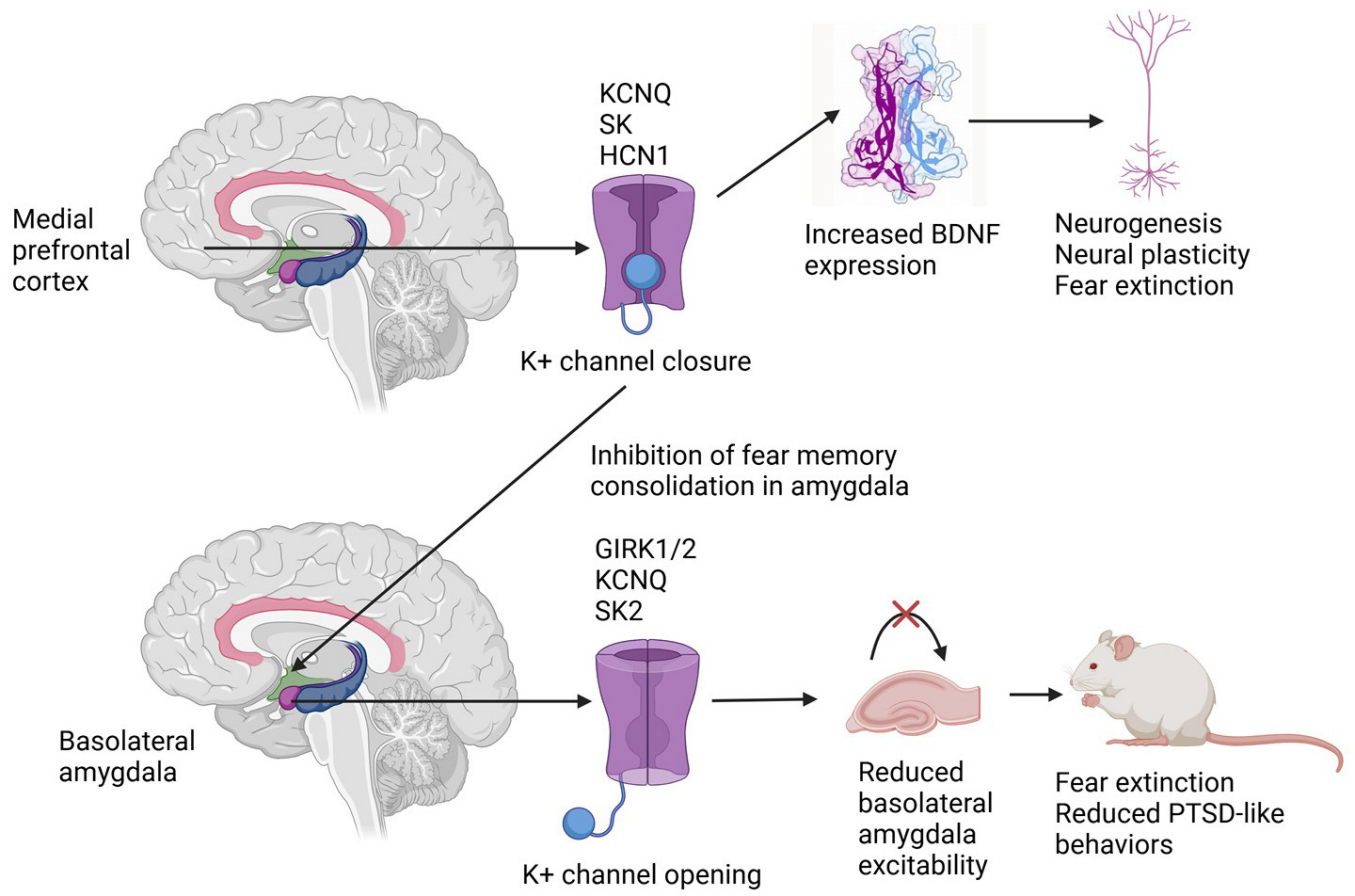
Differential effects of potassium channel subtype activation in the medial prefrontal cortex and the basolateral amygdala in rodent models of post-traumatic stress disorder. See the text for explanations of potassium channel nomenclature.

The evidence reviewed above is summarized in Figure 1 below.

## Limitations of the available evidence

The realistic appraisal of the evidence presented above requires an acknowledgment of both conceptual and methodological limitations. First, it is not clear to what extent animal models of PTSD, such as SPS and SPS&S, truly overlap with the syndrome of PTSD in trauma-exposed humans. Second, findings of K^+^ channel alterations in murine and primate brains may not “translate” directly to human brain physiology: therefore, these results require replication in humans. Third, the relationship between K^+^ channel activity and PTSD-like phenomena is complex, and depends crucially on factors such as the specific model of PTSD, the species being studied, the brain region being studied, and the pharmacological agents used to activate or block specific K^+^ channels. Finally, it is likely that specific K^+^ channel subtypes represent only one of many molecular mechanisms involved in PTSD, and that their activity depends crucially on levels of specific neurotransmitters, hormones, and even immune-inflammatory regulatory proteins. These limitations highlight the need for research on altered K^+^ channel functioning in humans with PTSD and other disorders related to traumatic stress.

## Summary and conclusions

The evidence available from animal models suggests that K+ channels from several families—voltage-gated, inward rectifying and calcium-activated—could play a role in the pathogenesis of PTSD-like phenomena following exposure to traumatic stress. At the cortical level, K^+^ channel activation may maintain PTSD symptoms by interfering with neural plasticity and fear extinction; at the limbic level, and particularly in specific regions of the amygdala, K^+^ channel activation may help in fear extinction and reduce PTSD symptoms. Pharmacological therapies aimed at “balancing” or “stabilizing” K^+^ channel activity between these brain regions may offer advantages over existing treatments for PTSD, and it is possible that some emerging treatments for this disorder, such as ketamine and prazosin, may act partly through their effects on K^+^ flux through specific channel types. The development of clinically effective and safe activators or antagonists of these channel subtypes may represent a significant step forward in the management of this chronic and disabling condition.

## Author contributions

RR: Conceptualization, Formal analysis, Methodology, Visualization, Writing – original draft, Writing – review & editing.
